# Distal forearm squeeze test for the diagnosis of digital flexor tendon injuries

**DOI:** 10.1186/s12891-023-07104-3

**Published:** 2023-12-16

**Authors:** Yunlong Zhi, Chengyue Wu, Maoqiang Li

**Affiliations:** 1 Department of Orthopaedic Surgery, Affiliated Hangzhou First People’s Hospital, Zhejiang University School of Medicine, Hangzhou, Zhejiang China; 2 The Fourth Clinical Medical College, Zhejiang Chinese Medical University, Hangzhou, Zhejiang China

**Keywords:** Flexor tendon injuries, Distal forearm, Diagnosis

## Abstract

**Background:**

The forearm/wrist squeeze/compression test has been used to examine digital flexor tendon injuries with varied names. Furthermore, the test has not been minutely described and its mechanism remains unclear. We renamed the test the “distal forearm squeeze test”. The purpose of this study was to elaborate on the test and elucidate the mechanism.

**Methods:**

Two patients with digital flexor tendons ruptured in zone 3 and zone 1 respectively and 50 outpatients with intact digital tendons underwent the test. Then the test was performed on 3 chickens under 4 conditions. First, when the digital flexor and extensor tendons were all intact. Second, after the flexor tendons of the third toe were transected. Third, after the flexor tendons of all toes of the foot were transected. Finally, after the flexor and extensor tendons of all toes of the foot were transected.

**Results:**

In the patient with digital flexor tendons ruptured in zone 3, the test showed that the injured digit was flexed slightly while the uninjured digits were flexed obviously. In the patient with digital flexor tendon ruptured in zone 1, after separate stabilization of the proximal interphalangeal (PIP) joints of the injured and uninjured fingers in extension, the test showed that the distal interphalangeal joint of the patient’s injured finger had no response, while those of the uninjured fingers were flexed. All 50 subjects showed clenched or half-clenched hands in response to the test. The test showed that all toes were flexed when the digital tendons of the chicken were intact. All toes were flexed except the third toe after the flexor tendons of the third toe were transected. All toes were extended after all the digital flexor tendons were transected. All toes had no response after all the digital flexor and extensor tendons were transected.

**Conclusions:**

The distal forearm squeeze test is valuable in examining digital flexor tendon injuries. If only the flexor digitorum profundus tendon is examined, the PIP joint of the finger should be stabilized in extension during the test.

## Background

Hand injuries are extremely common in clinical practice [1–5]. Among hand injuries, digital flexor tendon injuries are a challenging and demanding problem [6, 7], although the treatment has improved greatly in recent decades [8, 9]. In addition to taking the history and observing the cascade of the digits, digital flexor tendon injuries are often checked by asking the patient to actively flex the fingers [10]. If the patient is unable or unwilling to cooperate, such as in coma, with mental disorders, in early childhood, etc., active finger flexion is difficult. For example, the diagnosis of digital flexor tendon injuries in young children is difficult and often delayed compared with that in adults [11]. There are many reasons for this phenomenon, and lack of cooperation from young children is an important one.

The forearm squeeze/compression test has been used to test the integrity of digital flexor tendons [12, 13]. It was muscles or muscle bellies in the forearm that were squeezed in previous studies [13, 14]. In our experience, normally, squeezing flexor muscles or muscle bellies in the forearm will not produce obvious flexion of digits and even produce extension. Instead, zone 5 of the flexor tendons or flexor musculotendinous junctions in the forearm were squeezed in this study. It is possible that the distal forearm (zone 5 of the flexor tendons or flexor musculotendinous junctions) was squeezed in previous studies, but the statement was not very exact. To better understand and perform the test, we renamed the test the “distal forearm squeeze test” in this study. Furthermore, the test has not been described in detail, and its mechanism has not been elucidated. The purpose of this study was to fill the voids. Specifically, the purpose was to answer the following questions: (1) which part of the forearm should be squeezed in the test; (2) if both the flexor digitorum superficialis (FDS) and flexor digitorum profundus (FDP) tendons of a finger were ruptured, what’s the performance of the finger in response to the test; (3) if all the digital flexor tendons of a hand were ruptured, what’s the performance of the hand in response to the test; (4) if all the digital flexor tendons of a hand were ruptured, can the test be used to examine the digital extensor tendon injuries; (5) what’s the relationship between the digital flexor tendons and the extensor tendons in the test; (6) can the test be used to examine isolated FDS or FDP tendon injuries and if it is can, how to perform the test; (7) if the similar test is performed in the lower extremity, can the test be used to examine the flexor tendon injuries of the foot; and (8) what’s the mechanism of the test.

## Methods

### Clinical experiment

The ethics approval of the clinical experiment was waived by the Ethics Committee of Affiliated Hangzhou First People’s Hospital, Zhejiang University School of Medicine, considering there was neither harm to the subjects nor sensitive personal information of the subjects. From April 2022 to July 2023, 2 patients with digital flexor tendon ruptures were enrolled in the study. One patient was a 28-year-old male, whose both FDS and FDP tendons of the left index finger were severed by glass in zone 3. The other patient was a 74-year-old male, whose FDP tendon of the left index finger was severed by sharp metal in zone 1. For the young patient, the distal forearm squeeze test was performed before and after tenorrhaphy. For the old patient, before tenorrhaphy, the distal forearm squeeze test was performed after separate stabilization of the proximal interphalangeal (PIP) joints of the injured and uninjured fingers of the patient’s left hand in extension. After tenorrhaphy, the test was performed in the same manner.

Meanwhile, 50 outpatients without a history of extremity diseases were randomly selected to participate in the study. They were 26 males and 24 females, with an average age of 36.8 years (range, 2 to 93 years). Each subject underwent a bilateral distal forearm squeeze test. Our method of the test was placing one hand of the examiner around the distal half of the forearm of the examinee and squeezing. During the test, the thumb of the hand of examiner was opposite to the 4 fingers, so the hand was in a circular shape, and the examined hand was relaxed. Meanwhile, the performance of the examined hand was observed. In addition, among them, 5 subjects were randomly selected to undergo a bilateral distal forearm squeeze test after the PIP joints of the fingers and the metacarpophalangeal (MP) joints of the thumbs were stabilized in extension respectively. Specifically, stabilizing the PIP joint of the finger or the MP joint of the thumb of the examinee in extension with one hand of the examiner, placing the other hand around the distal half of the forearm of the examinee in the abovementioned circular shape and squeezing. The examined hand was relaxed during the test. Meanwhile, the performance of the examined hand was observed. Then the 5 subjects underwent a bilateral distal shank squeeze test, and the method was similar to that of the distal forearm squeeze test. Specifically, placing one hand of the examiner around the distal shank of the examinee in the abovementioned circular shape and squeezing. The examined foot was relaxed during the test. Meanwhile, the performance of the examined foot was observed. They were 2 males and 3 females, with an average age of 32.6 years (range, 19 to 51 years). All 2 injured patients and the 50 uninjured subjects or their legal guardians gave their oral informed consent to participate in the study.

### Animal experiment

**Fig. 1:**
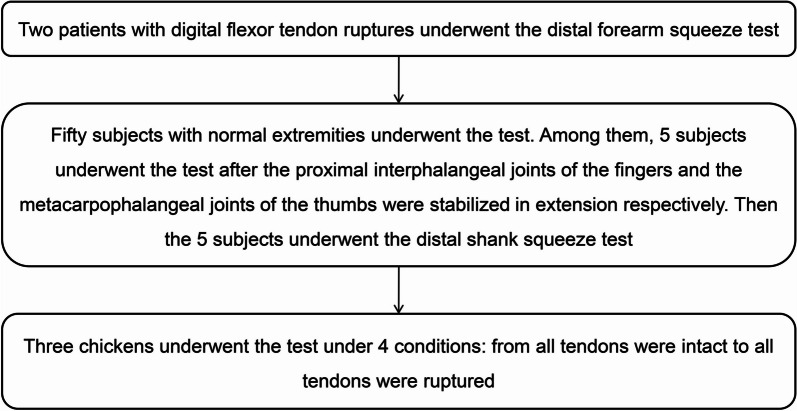
The flowchart

The animal experiment was approved by the Zhejiang University Institutional Animal Care and Use Committee (approval No. ZJU20230304). Three skeletally mature male Sanhuang chickens weighing 1.8 to 2.1 kg each without a history of leg diseases were selected for the experiment. After the chickens were sacrificed by intramuscular injection of an overdose of pentobarbital sodium and skin preparation, the bilateral distal forearm/shank squeeze test was performed under the following 4 conditions. First, when the digital flexor and extensor tendons were all intact, meaning that nothing was done to the tendons; second, after the flexor tendons of the third toe (the longest toe) were transected completely at the foot level with a scalpel; third, after the flexor tendons of all toes of the foot were transected completely at the foot level with a scalpel; and finally, after the flexor and extensor tendons of all toes of the foot were transected completely at the foot level with a scalpel. The method of the test was placing one hand of the examiner around the distal shank of the chicken in the abovementioned circular shape and squeezing. Meanwhile, the performance of the examined foot was observed. The flowchart was shown in Fig. 1.

## Results

### Clinical experiment

**Fig. 2:**
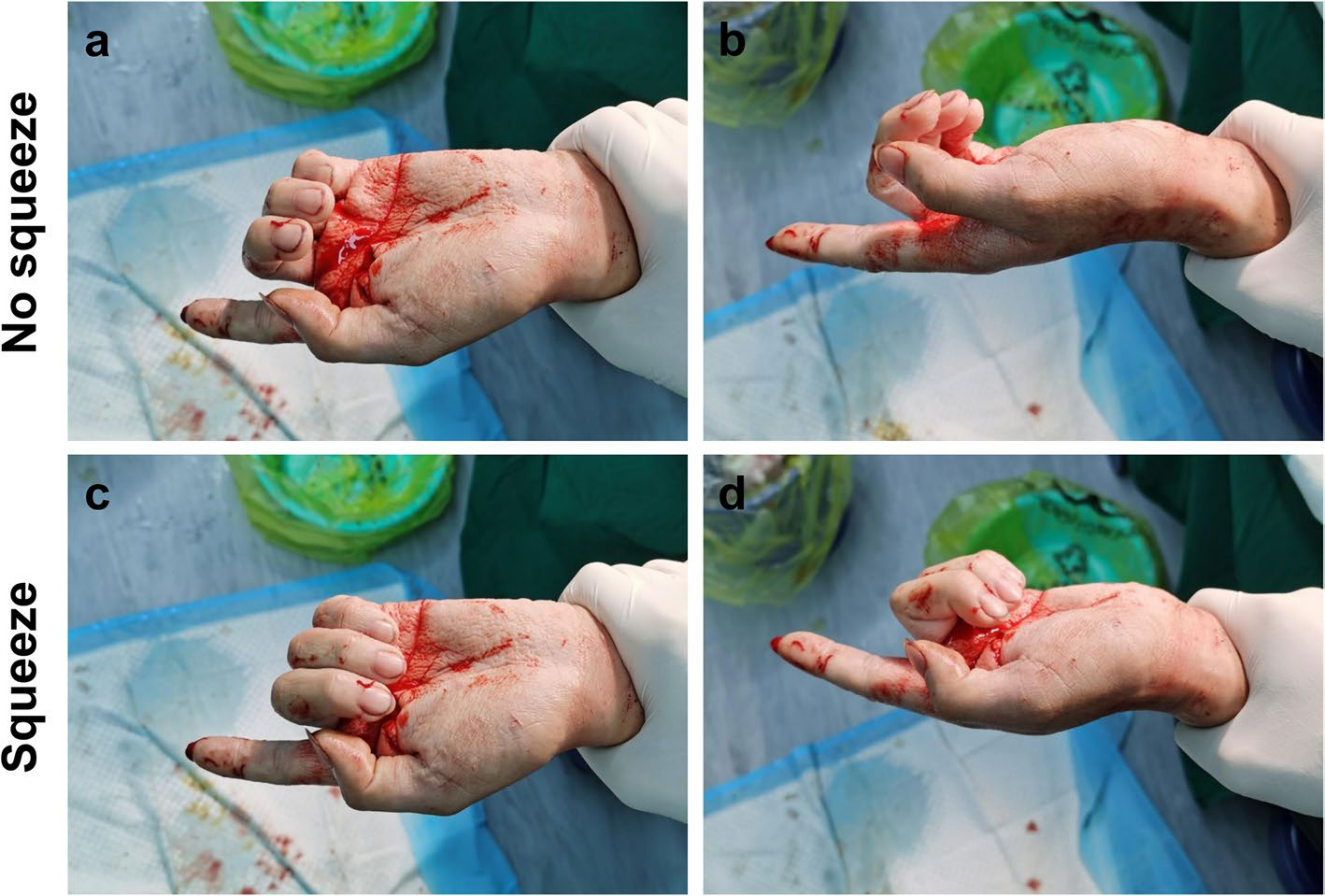
The manifestations of the injured young patient in the distal forearm squeeze test. **a** and **b** The hand was relaxed. **c** and **d** The injured digit was in slight flexion while the uninjured digits were in obvious flexion

**Fig. 3:**
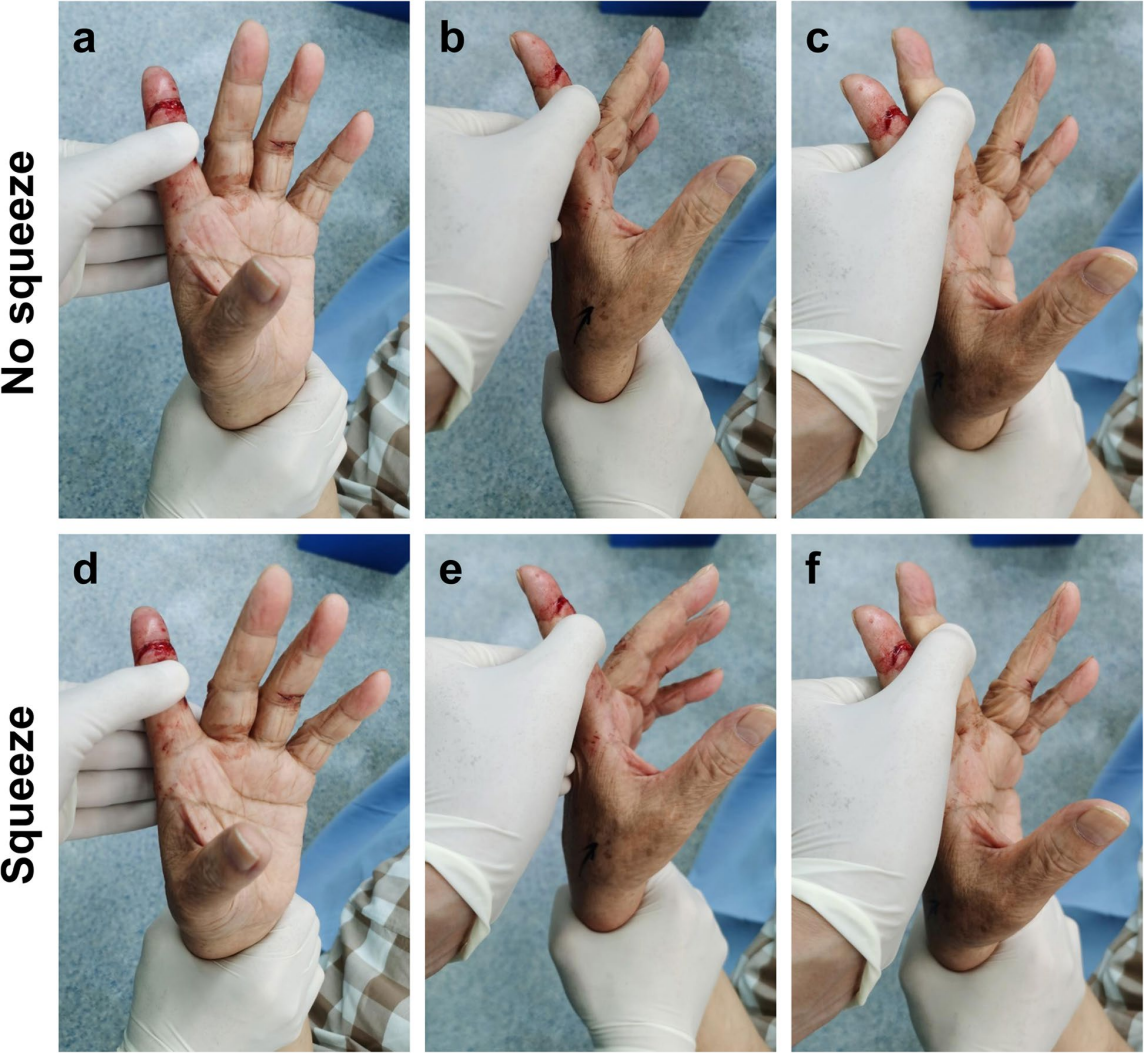
The manifestations of the injured old patient in the profundus distal forearm squeeze test. **a**, **b**, **d** and **e** The DIP joint of the injured finger had no response. **c** and **f** The DIP joints of the uninjured fingers were in flexion

In the young patient with both FDS and FDP tendons of the left index finger ruptured in zone 3, the distal forearm squeeze test showed that the injured digit was flexed slightly while the uninjured digits were flexed obviously (Fig. 2). After tenorrhaphy, the injured digit was flexed obviously in response to the test. In the old patient with isolated FDP tendon of the left index finger ruptured in zone 1, when the PIP joints of the left hand were stabilized in extension respectively, the test showed that the distal interphalangeal (DIP) joint of the injured finger had no response (Fig. 3a, b, d and e), while those of the uninjured fingers were in flexion (Fig. 3c and f). After tenorrhaphy, the test performed in the same manner showed that the DIP joint of the injured finger returned to flexion, similar to the uninjured fingers.

**Fig. 4:**
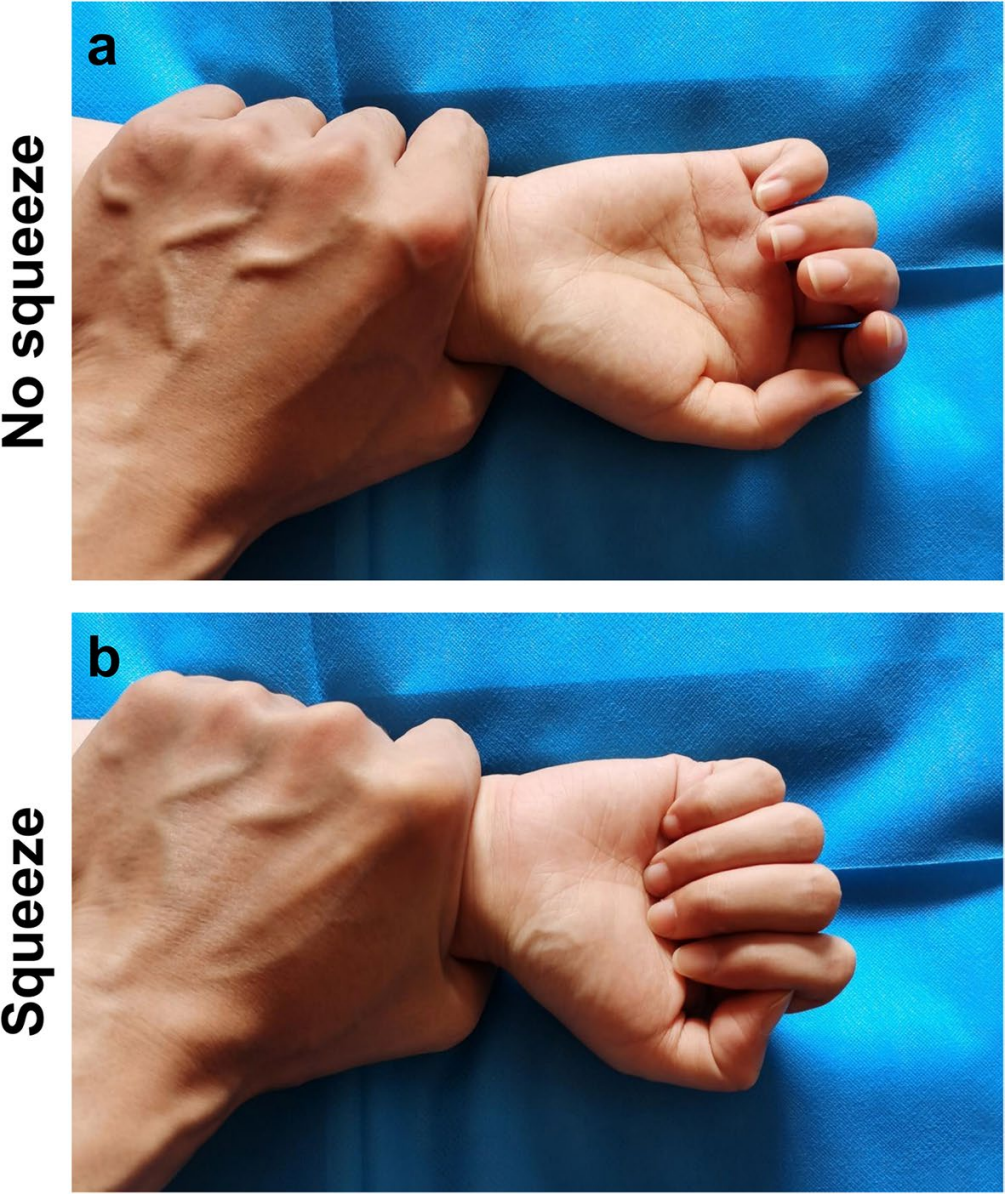
The hands of the uninjured subjects were clenched or half-clenched in the distal forearm squeeze test

A total of 100 hands of 50 uninjured subjects were all distal forearm squeeze test negative, being clenched or half-clenched when the distal forearms were squeezed (Fig. 4).

**Fig. 5:**
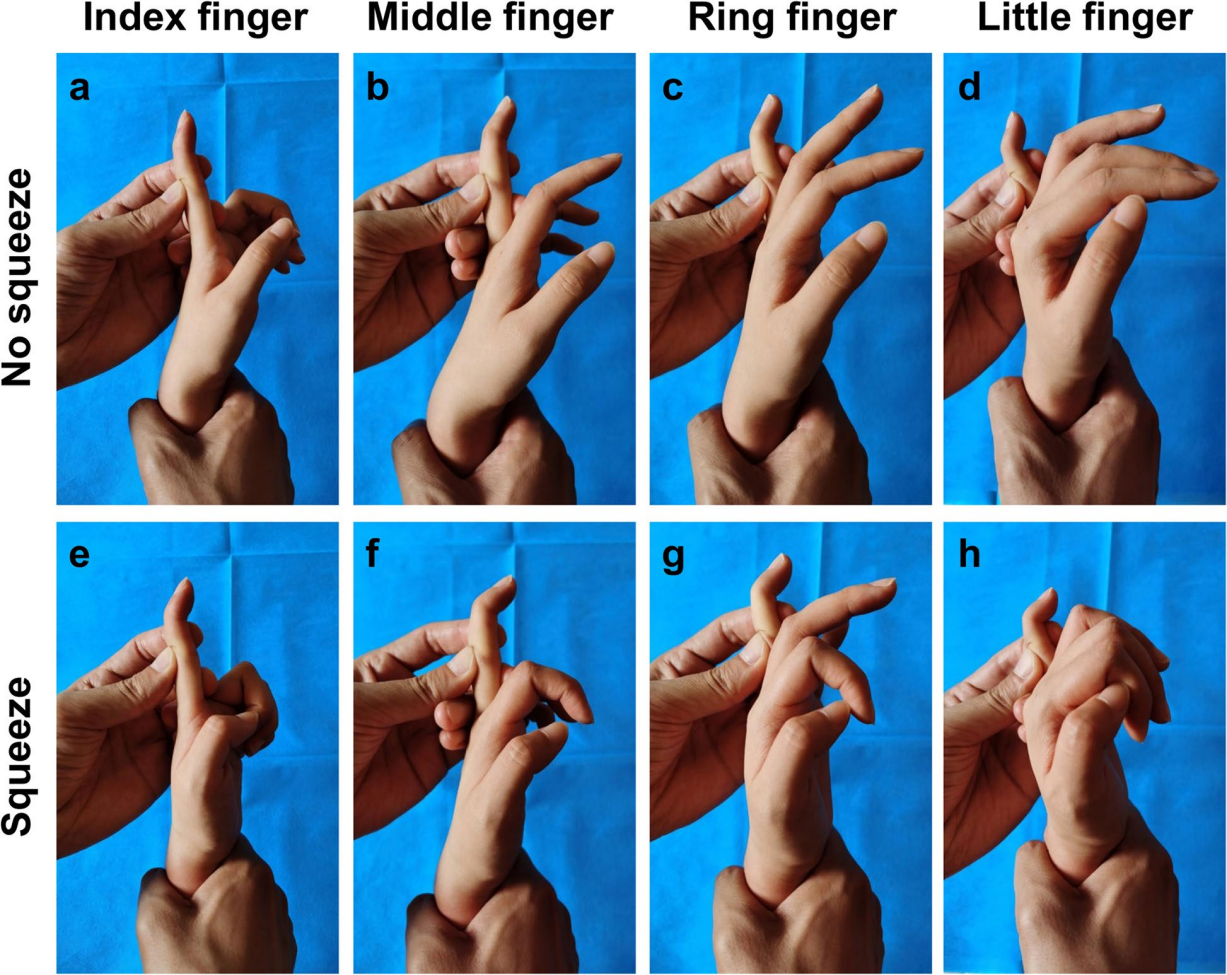
The DIP joints of the uninjured subjects were flexed in the profundus distal forearm squeeze test. **a** and **e** Index finger. **b** and **f** Middle finger. **c** and **g** Ring finger. **d** and **h** Little finger

**Fig. 6:**
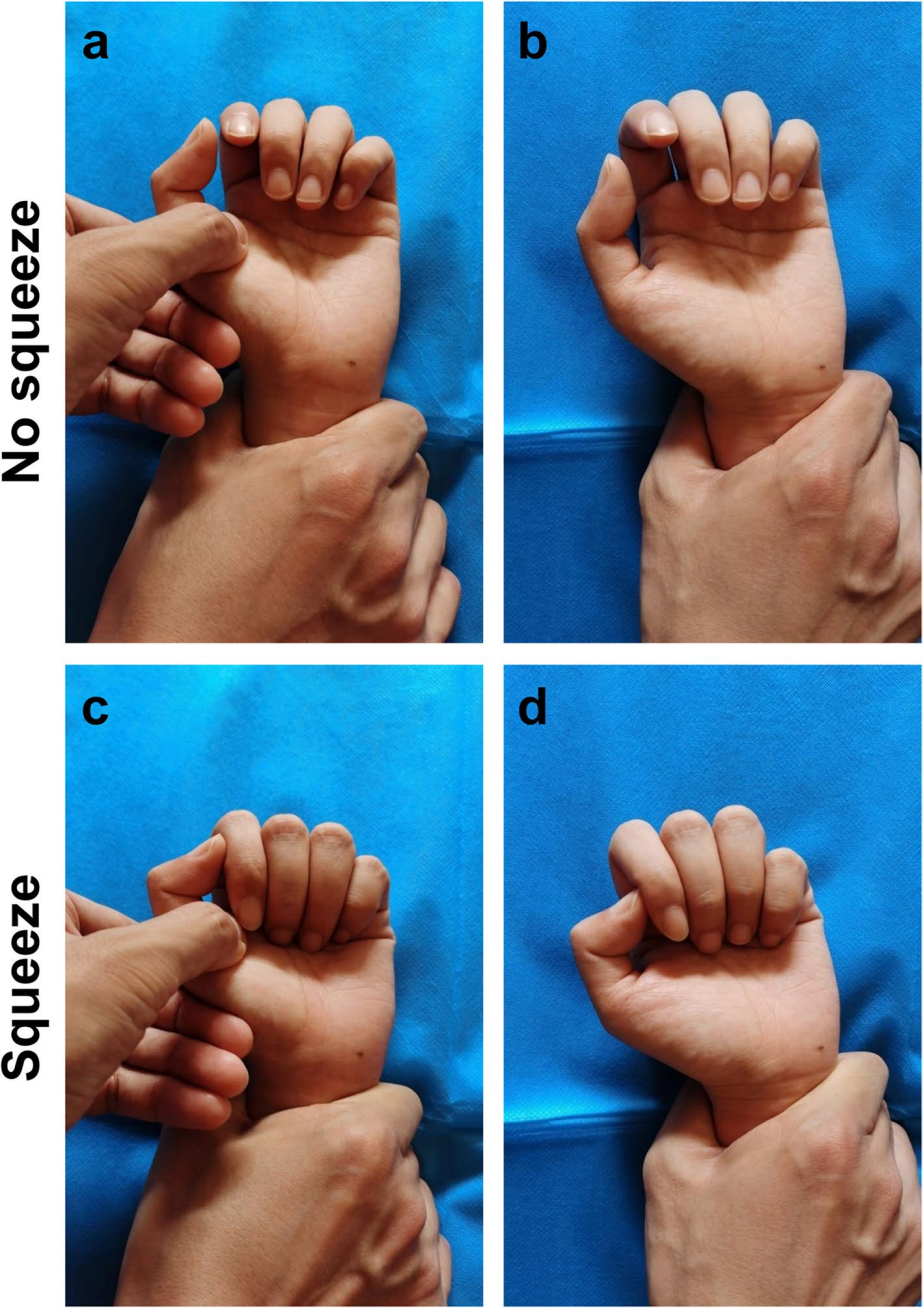
The interphalangeal joints of healthy thumbs were flexed in the distal forearm squeeze test. **a** and **c** Stabilizing the thumb MP joint in extension. **b** and **d** Without stabilizing the thumb MP joint in extension

**Fig. 7:**
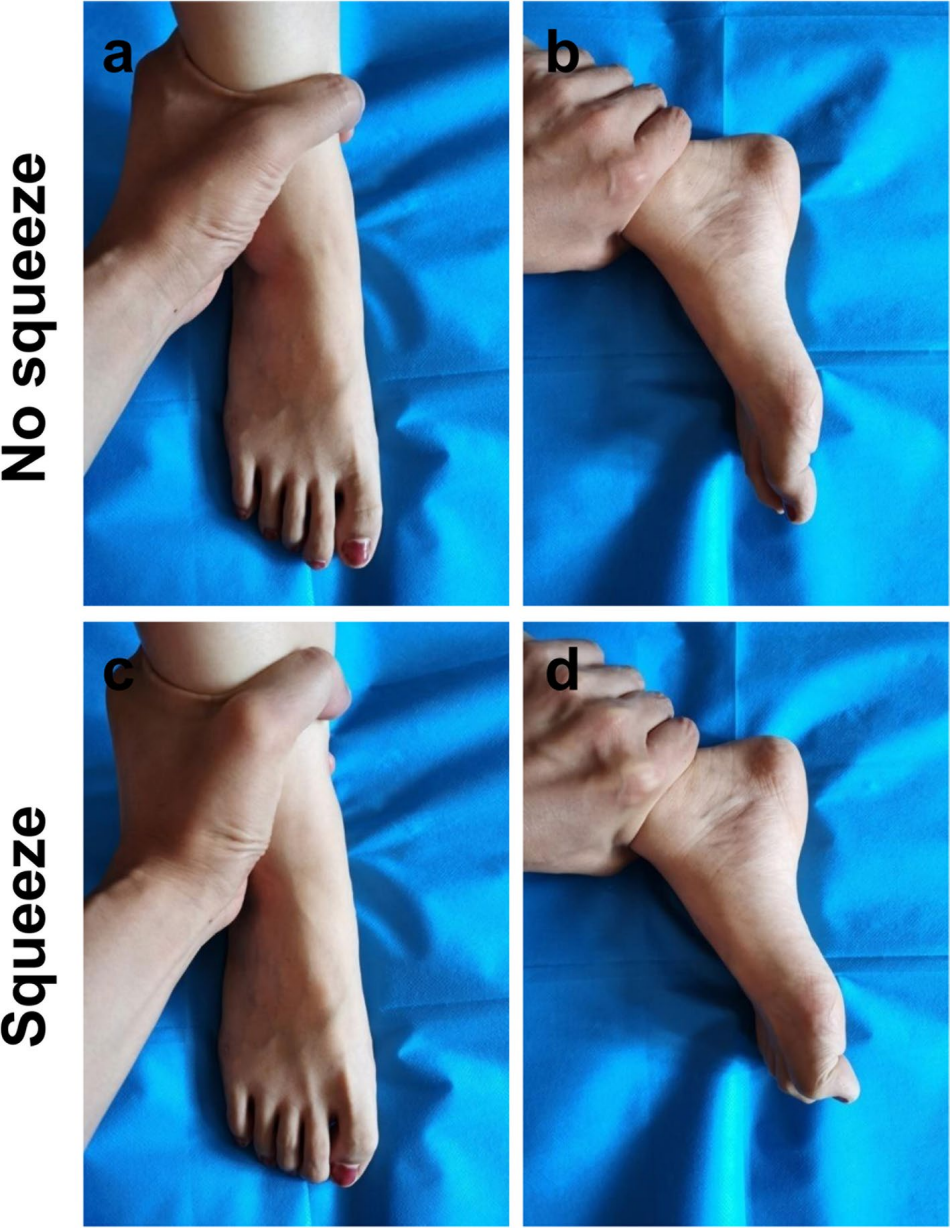
All toes of the uninjured subjects were flexed in the distal shank squeeze test

In the 5 subjects who underwent the bilateral distal forearm squeeze test after the PIP joints of the fingers and the MP joints of the thumbs were stabilized in extension respectively, the test showed that the DIP joints of the fingers and the interphalangeal joints of the thumbs were flexed (Figs. 5 and 6). A total of 10 feet of the 5 subjects showed plantar flexion of each toe in response to the distal shank squeeze test (Fig. 7).

### Animal experiment

**Fig. 8:**
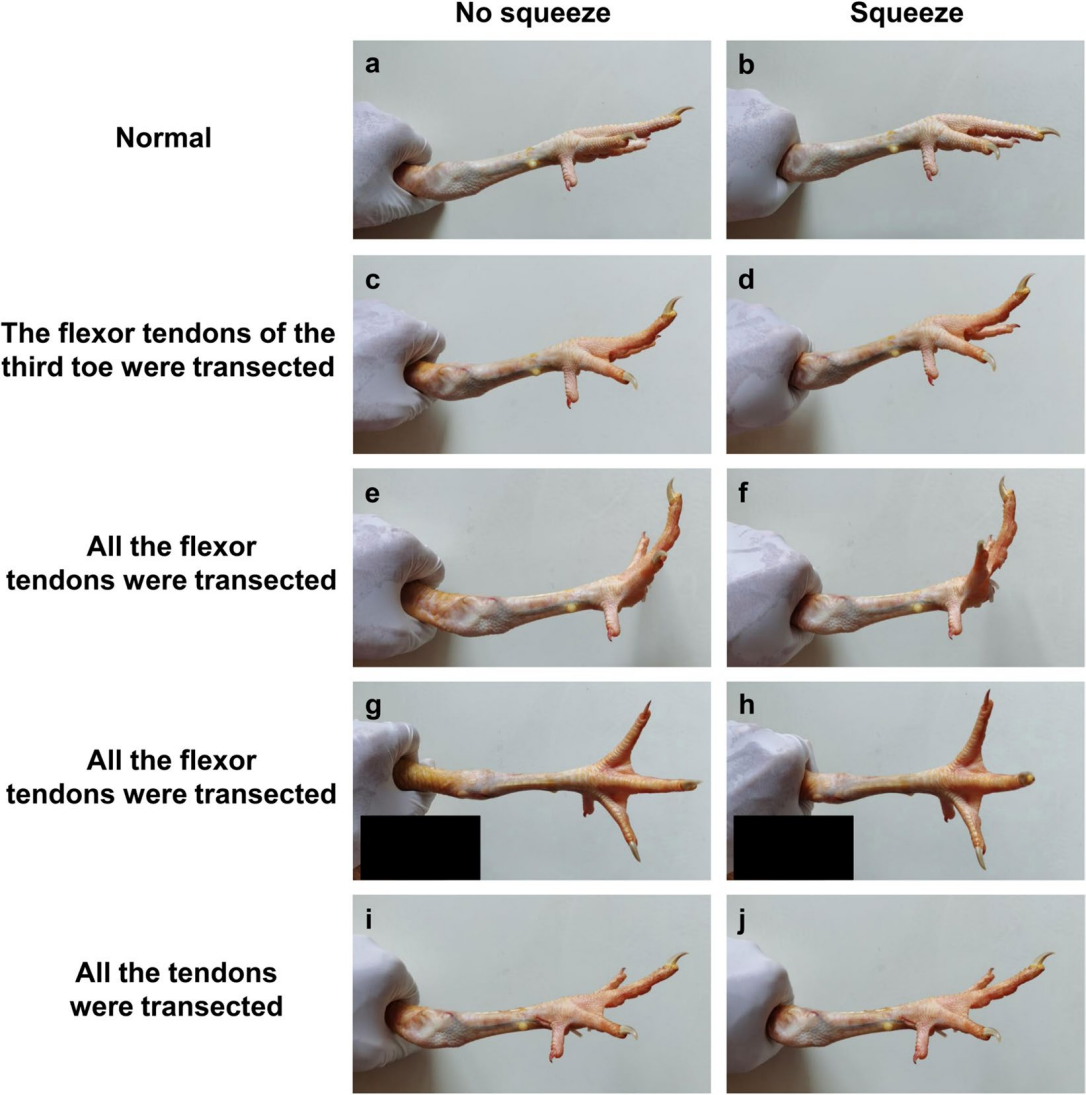
The manifestations of chickens in the distal forearm/shank squeeze test. **a** and **b** In the normal foot, all toes were in flexion. **c** and **d** After the flexor tendons of the third toe were transected, all toes were in flexion except the third toe. **e**, **f**, **g** and **h** After all the flexor tendons of the foot were transected, all toes were in extension. (i and j) After all the flexor and extensor tendons were transected, all toes had no response

When the digital flexor and extensor tendons of the chicken were all intact, the distal forearm/shank squeeze test showed that all toes were in flexion (Fig. 8a and b). After the flexor tendons of the third toe were transected completely, the test showed that all toes were in flexion except the third toe (Fig. 8c and d). After the flexor tendons of all toes of the foot were transected completely, the test showed that all toes were in extension (Fig. 8e, f, g and h). After the flexor and extensor tendons of all toes of the foot were transected completely, the test showed that all toes had no response, neither flexion nor extension (Fig. 8i and j).

## Discussion

When one’s distal forearm is squeezed, the hand in the rest position will appear clenched or half-clenched, which must be caused by the proximal movement of the squeezed digital flexor tendons. Therefore, if the flexor tendons of a finger are ruptured, in theory, the finger will not be flexed in response to the distal forearm squeeze test, at least not as obviously as the uninjured fingers. As a result, we can check whether the digital flexor tendons are ruptured with the test.

**Fig. 9:**
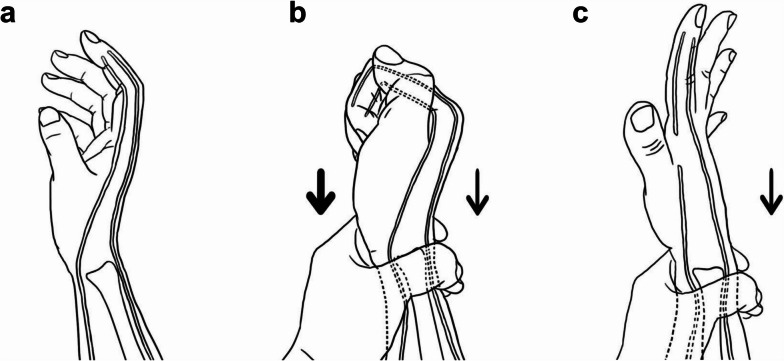
The mechanism of the distal forearm squeeze test. **a** The normal hand. **b** The digital flexor and extensor tendons are in a tug-of-war during the distal forearm squeeze test. In normal conditions, the digital flexor tendons win by overwhelming advantage. **c** If the digital flexor tendons are ruptured, the extensor tendons win. The thick arrow represents the force of digital flexor tendons, while the thin arrow represents the force of digital extensor tendons

The flexor and extensor tendons in the distal forearm are superficial. When the distal forearm is squeezed, the flexor and extensor tendons are pressed towards the radius and ulna, resulting in the movement trend of the distal ends of the tendons towards the proximal ends. The thickness of the volar side of the distal forearm is greater than that of the dorsal side, making the passive displacement trend of the distal flexor tendons greater than that of the distal extensor tendons. Meanwhile, the muscle strength of flexors is greater than that of extensors. As a result, in normal conditions, the hand is clenched or half-clenched when the distal forearm is squeezed (Fig. 9b). If both the FDS and FDP tendons of one finger of a patient are ruptured and the influence of other fingers is not considered, the finger is extended in response to the distal forearm squeeze test because the finger is pulled by the extensor digitorum tendon (Fig. 9c). This is a positive distal forearm squeeze test. If the flexor and extensor tendons of one finger of a patient are all ruptured and the influence of other fingers is not considered, the finger has no response to the test. This is also a positive distal forearm squeeze test. In fact, the flexion of the healthy fingers will produce weak flexion of the finger with ruptured FDS and FDP tendons during the test due to force transmission through the digital webs. In short, the normal response of a finger to the test is a large degree of flexion. If the flexion degree of a finger is not as large as that of the healthy finger, with no response, or even extension, the finger is distal forearm squeeze test positive, suggesting flexor tendon lacerations.

If only the FDP tendon is examined, the PIP joint of the finger should be stabilized in extension during the test to exclude the influence of the FDS tendon. Considering that such a distal forearm squeeze test is only used to examine FDP tendon injuries, we named it the “profundus distal forearm squeeze test”. In normal conditions, the DIP joint is flexed. If the DIP joint does not respond or is even extended, the finger is profundus distal forearm squeeze test positive, proving that the FDP tendon is ruptured. When the flexor pollicis longus (FPL) tendon is examined, it’s both feasible to stabilize the thumb MP joint in extension or not during the test (Fig. 6). This is because during the test, among all the tendons and muscles that can flex the thumb, only the FPL tendon is squeezed and moves proximally, while the flexor pollicis brevis, the adductor pollicis and their tendons are not squeezed. As a result, it is not necessary to rule out their influence on thumb flexion.

During the study period, we treated open injuries of the extensor tendons of 3 digits in 3 patients, including 1 extensor pollicis longus tendon and 1 extensor tendon of the index finger and ring finger respectively. The affected digit of the patient with a ruptured extensor tendon showed the same degree of flexion as the healthy digits in response to the distal forearm squeeze test. This demonstrated that the influence of the digital extensor tendons on the test was negligible when the digital flexor tendons were intact. However, when the digital flexor tendons were ruptured, the influence of the digital extensor tendons on the test was shown.

Before the study period, we encountered a patient whose flexor tendons of 5 digits in one hand were all ruptured in an industrial accident, while the digital extensor tendons were all intact. The distal forearm squeeze test showed that all 5 digits of the hand were extended (Fig. 9c), which was consistent with the animal experiment (Fig. 8e, f, g and h). This indicated that the test also worked in examining the integrity of digital extensor tendons in case both the FDS and FDP tendons were ruptured. Of course, this needed to eliminate the interference of healthy finger flexion on the affected finger during the test.

The digital flexor and extensor tendons are in a tug-of-war during the distal forearm squeeze test. In normal conditions, the digital flexor tendons win by overwhelming advantage. Once the digital flexor tendons are ruptured, the extensor tendons win (Fig. 9).

To the best of our knowledge, this is the first study to describe the distal forearm squeeze test in detail and elucidate its mechanism, although the forearm squeeze/compression test was mentioned before [12, 13]. It was muscles or muscle bellies in the forearm that were squeezed in previous studies [13, 14], while it was zone 5 of the flexor tendons or flexor musculotendinous junctions in the forearm that were squeezed in our study. In our experience, in normal conditions, squeezing flexor muscles or muscle bellies in the forearm will not elicit significant flexion of digits and even elicit extension. We consider that when muscles or muscle bellies in the forearm are squeezed, they become thinner and longer, causing their tendons to move distally. At the same time, the pulling force of the extensors results in the extension of digits.

Compared with the “forearm squeeze/compression test”, the previously mentioned “wrist squeeze test” [15] is more appropriate to describe the test. However, the scope of the wrist is not large enough. In our experience, squeezing the distal half of the forearm can produce digit flexion. By comprehensive consideration, we renamed the test the “distal forearm squeeze test”.

The human hand is just large enough to hold the distal forearm, and the distal forearm squeeze test can be performed in almost any posture of the patient, making the test simple. The distal forearm squeeze test does not need cooperation from the patient, so the test still works in patients under general anesthesia or brachial plexus anesthesia. After tenorrhaphy, the repair effect can also be checked immediately by the test.

The essence of the distal forearm squeeze test is the passive movement of the digital flexor tendons towards the proximal end due to the squeeze force (Fig. 9). The degree of passive digit flexion of a patient through the distal forearm squeeze test is close to that of active digit flexion. When the digital flexor tendons are ruptured, the manifestation during the test is similar to that of asking the patient to actively flex the digits. Therefore, the distal forearm squeeze test cannot be used to examine isolated FDS tendon rupture, which is a disadvantage. The test is most suitable for patients with both FDS and FDP tendon ruptures. In patients with digital flexor tendon ruptures, ruptures of both FDS and FDP tendons were the most common [16]. Furthermore, if the patient’s PIP joint is stabilized in extension, the test can be used to examine isolated FDP tendon rupture. Therefore, the distal forearm squeeze test is practical in the diagnosis of digital flexor tendon ruptures.

The human foot is similar to the hand, while the manifestation and mechanism of the distal shank squeeze test is similar to that of the distal forearm squeeze test. The distal shank squeeze test can be used to examine flexor tendon injuries of the foot (Fig. 7).

The distal forearm/shank squeeze test is similar to the Simmonds-Thompson test [14, 17, 18], both of which produce movement in the more distal extremity through force transmission [19]. The difference is that it is muscle bellies that are squeezed in the Simmonds-Thompson test, while it is tendons or musculotendinous junctions that are squeezed in the distal forearm/shank squeeze test.

Early treatment of digital flexor tendon injuries is easy, but late treatment is troublesome, so prompt diagnosis is important. Open digital flexor tendon injuries are less likely to be missed because of the necessity for emergency surgery exploration, whereas closed injuries, such as avulsion of the FDP tendon from insertion at the distal phalanx, which are relatively common in athletes, are more likely to be missed [20–22]. It is hoped that the distal forearm squeeze test will be of value in making timely diagnosis of digital flexor tendon injuries.

During the distal forearm squeeze test, all 100 hands of 50 uninjured subjects appeared clenched or half-clenched. The age span of the 50 subjects was very large, ranging from young children to the elderly, demonstrating the universality of the test.

The primary limitation of this study is the small number of patients with digital flexor tendon injuries. We had only 2 such patients during the 16-month study period. Another limitation is that we have not quantified the flexion degree of the digits in response to the test, because we think it feasible to judge if the flexion degree of a digit is normal by comparing the flexion degree of the injured digit with that of uninjured digit.

## Conclusions

The distal forearm squeeze test can be used to examine digital flexor tendon injuries. If only the FDP tendon is examined, the PIP joint of the finger should be stabilized in extension during the test. When the FPL tendon is examined, it’s both feasible to stabilize the thumb MP joint in extension or not during the test. The test is especially suitable for unconscious or uncooperative patients. The mechanism of the test is the passive movement of the digital flexor tendons towards the proximal end due to the squeeze force.

## Data Availability

The datasets used and analysed during the current study are available from the corresponding author on reasonable request.

## Abbreviations

DIP: Distal interphalangeal
FDP: Flexor digitorum profundus
FDS: Flexor digitorum superficialis
FPL: Flexor pollicis longus
MP: Metacarpophalangeal
PIP: Proximal interphalangeal
